# Supplementary material for: Biosynthesis of Gold Nanostructures and Their Virucidal Activity Against Influenza A Virus
Source: Int J Mol Sci. 2025 Feb 24;26(5):1934. doi: 10.3390/ijms26051934 (PMC11899802; doi:10.3390/ijms26051934)
Supplement: Supplementary file 1 [file ijms-26-01934-s001.zip › ijms-3459216-supplementary.pdf]

## Supplementary Figures and Table

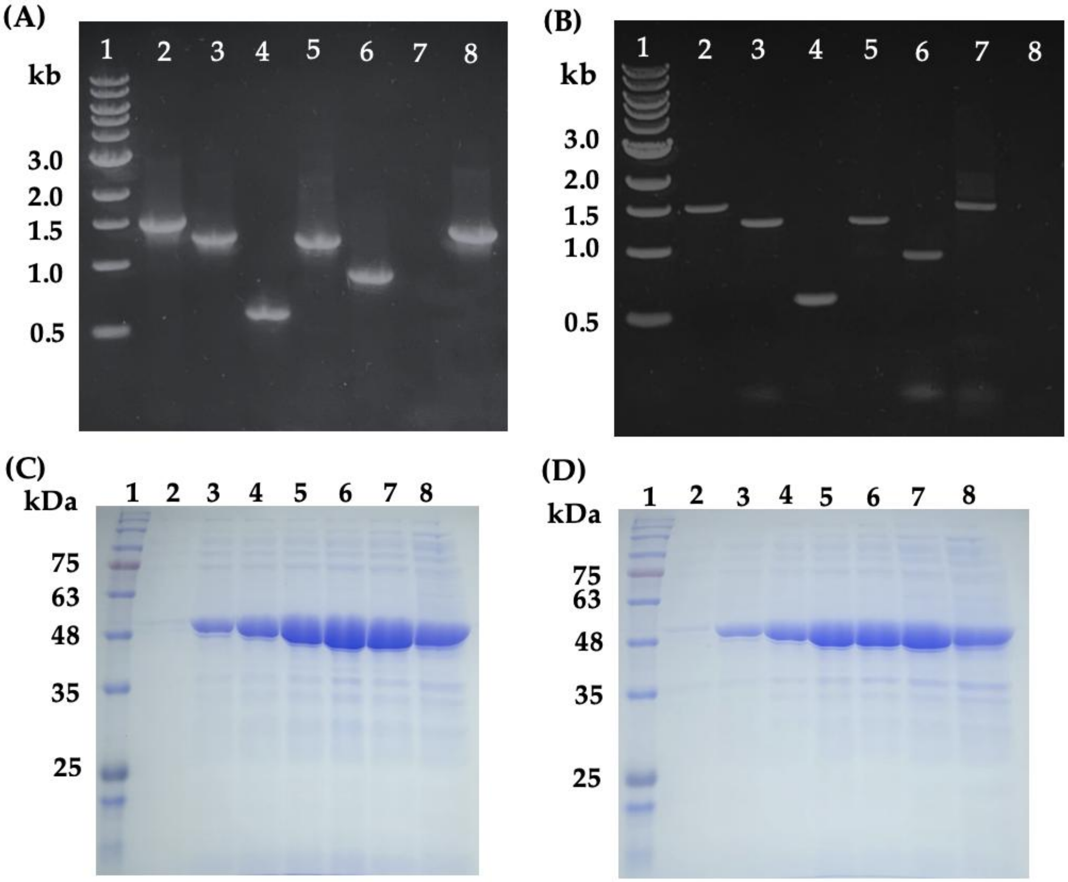


**Figure S1. Molecular and biochemical characterization of proteins with potential Au(III) reductase activity.** Agarose gel electrophoresis (1% w/v) of PCR products encoding proteins with potential Au(III) reductase activity, using **(A)** GoTar or **(B)** Q5 DNA polymerase. Lane 1: 1 kb DNA Ladder. Lanes 2-6: amplified products of *gor, cdr, oxi, trmFO,* and *trxB* genes. Lane 7: 16S rRNA gene (positive control). Lane 8: PCR negative control. **(C-D)** Representative SDS-PAGE analysis of gene expression induction and protein production for putative Au(III) reductase protein Gor. Lane 1: Molecular weight marker (AccuRuler RGB PLUS Prestained Protein Ladder). Lanes 2-8: Crude extracts of *E. coli* BL21(DE3) collected at 0, 1, 2, 4, 6, 8, and 24 hours post-induction. **(C)** Expression of Gor at 30°C. **(D)** Expression of Gor at 37°C.

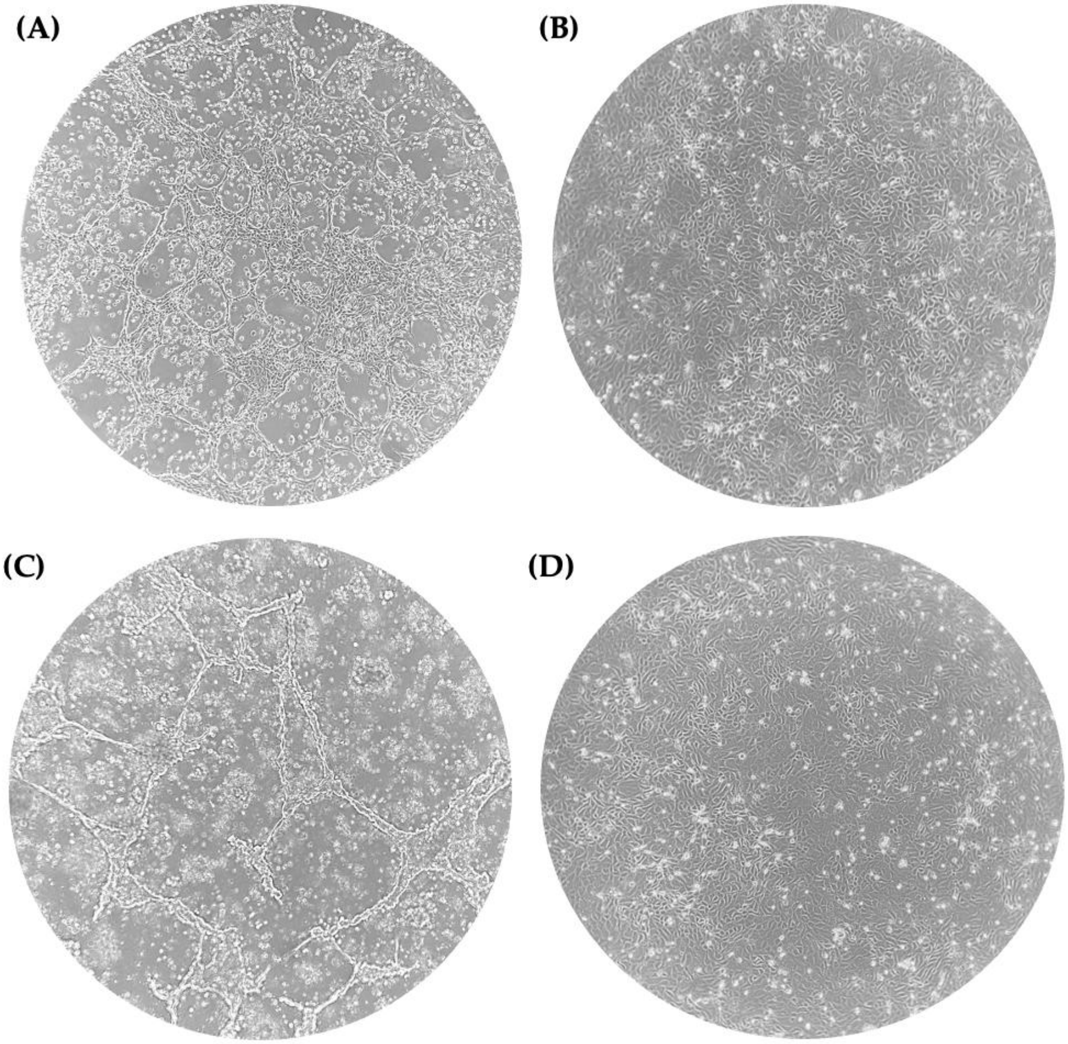


**Figure S2. Cytopathic effect in MDCK cell cultures infected with influenza A H1N1 virus.** MDCK cell cultures were infected with an MOI of 0.1 of influenza A H1N1 virus or maintained with 1X DPBS (uninfected control) for 1 hour. The cytopathic effect was observed at 48 **(A - B)** and 72 **(C - D)** hours post-infection in cultures infected with influenza A virus **(A and C)** and in uninfected control cultures **(B and D)**.

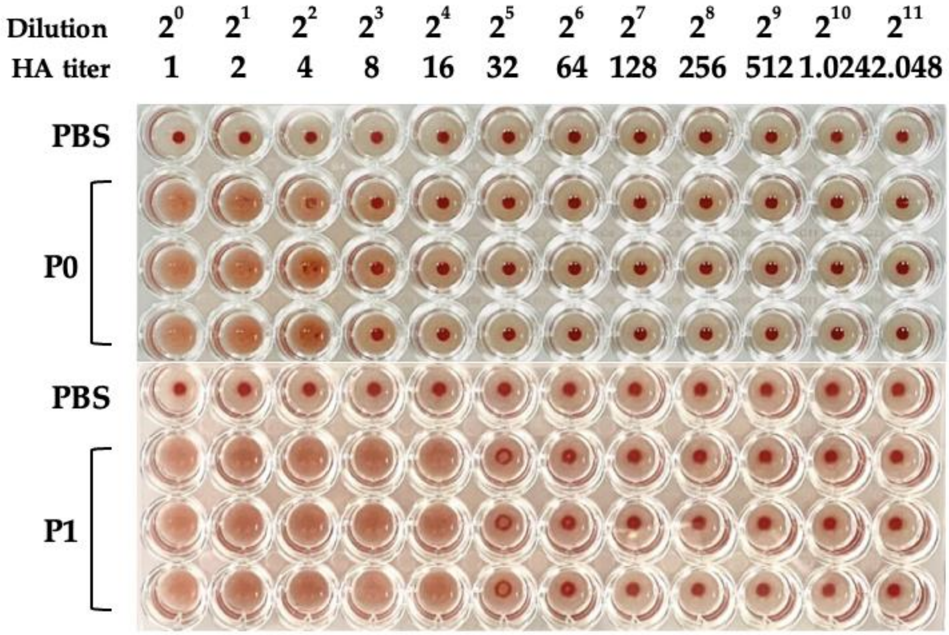


**Figure S3. Hemagglutination assay with supernatants collected post-infection.** The HA titer of influenza A virus was evaluated in two viral supernatant samples, P0 and P1, after 1:2 dilution in 1X PBS. 1X PBS was used as a negative control.

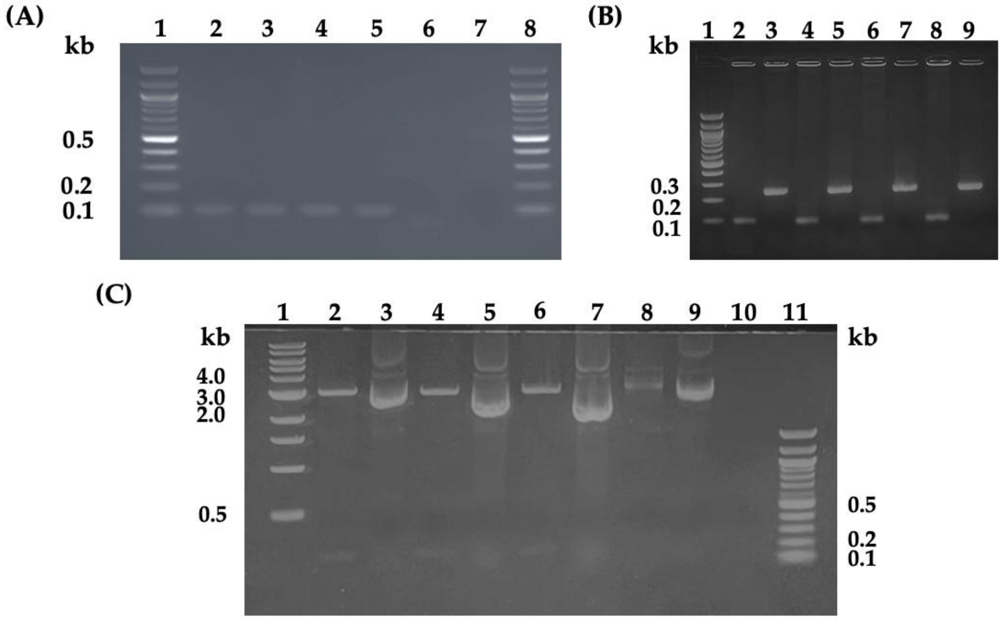


(D)

| | Description | Scientific Name | Max Score | Total Score | Query Cover | E value | Per. Ident | Acc. Len | Accession |
|---|---|---|---|---|---|---|---|---|---|
| ☑ | Influenza A virus (A/swine/France/22-130212/2013(H1N2)) segment 7 matrix protein 2 (M2) and matrix protein 1 (… | Influenza A virus … | 182 | 182 | 100% | 2e-41 | 99.01% | 1027 | KM267912.1 |
| ☑ | Influenza A virus (A/swine/Thailand/PB486/2009(H3N2)) segment 7 matrix protein 2 (M2) and matrix protein 1 (M1… | Influenza A virus … | 182 | 182 | 100% | 2e-41 | 99.01% | 1027 | KC859129.2 |
| ☑ | Influenza A virus (A/swine/Thailand/PB483/2009(H3N2)) segment 7 matrix protein 2 (M2) and matrix protein 1 (M1… | Influenza A virus … | 182 | 182 | 100% | 2e-41 | 99.01% | 1027 | KC841923.2 |
| ☑ | Influenza A virus (A/chicken/Egypt/096L-NLQP/2009(H5N1)) segment 7 matrix protein 2 (M2) and matrix protein 1… | Influenza A virus … | 182 | 182 | 100% | 2e-41 | 99.01% | 1027 | KR732404.1 |
| ☑ | Influenza A virus (A/swine/Thailand/PB485/2009(H3N2)) segment 7 matrix protein 2 (M2) and matrix protein 1 (M1… | Influenza A virus … | 182 | 182 | 100% | 2e-41 | 99.01% | 1014 | KC841931.1 |
| ☑ | Influenza A virus (A/swine/Thailand/CM480/2009(H3N2)) segment 7 matrix protein 2 (M2) and matrix protein 1 (M… | Influenza A virus … | 182 | 182 | 100% | 2e-41 | 99.01% | 1029 | KC859209.1 |
| ☑ | Influenza A virus (A/chicken/Hubei/wl/1997(H5N1)) segment 7, complete sequence | Influenza A virus … | 182 | 182 | 100% | 2e-41 | 99.01% | 1027 | DQ997138.1 |
| ☑ | Influenza A virus (A/chicken/Hubei/wf/2002(H5N1)) matrix protein 2 (M2) and matrix protein 1 (M1) genes, complet… | Influenza A virus … | 182 | 182 | 100% | 2e-41 | 99.01% | 1027 | DQ997086.1 |
| ☑ | Influenza A virus (A/Chicken/Henan/210/2004 (H5N1)) segment 7, complete sequence | Influenza A virus … | 182 | 182 | 100% | 2e-41 | 99.01% | 1027 | AY950238.1 |
| ☑ | Influenza A virus (A/Chicken/Pennsylvania/13552-1/98 (H7N2NSB)) matrix protein 1 (M1) and matrix protein 2 (M… | Influenza A virus … | 182 | 182 | 100% | 2e-41 | 99.01% | 1002 | AF073195.1 |

**Figure S4. Analysis of the conserved segment of the M gene using agarose gel electrophoresis and verification of insertion into the pGEM-T Easy vector. (A)** Agarose gel electrophoresis (2% w/v) of PCR-amplified products of the conserved segment of the M gene. Lanes 1 and 8: 100 bp DNA Ladder. Lanes 2-5: amplified products of the M gene conserved segment. Lanes 6 and 7: negative controls of the PCR reaction. **(B)** Verification of the insertion of the conserved M gene segment into the pGEM-T Easy vector. Lane 1: 100 bp DNA Ladder. Lanes 2-4-6-8: PCR products amplified with gene-specific primers. Lanes 3-5-7-9: PCR products amplified using one gene-specific primer and one vector-specific primer (M13R terminator). **(C)** Lane 1: 1 kb DNA Ladder. Lanes 2-4-6-8: *Eco*RI digestion of purified recombinant vectors from clones 1, 2, 3, and 4. Lanes 3-5-7-9: undigested purified recombinant vectors from clones 1, 2, 3, and 4. Lane 10: negative control for enzymatic digestion. Lane 11: 100 bp DNA Ladder. **(D)** The nucleotide sequence of the conserved M gene segment obtained after sequencing was used for a multiple alignment in the BLAST-N platform.

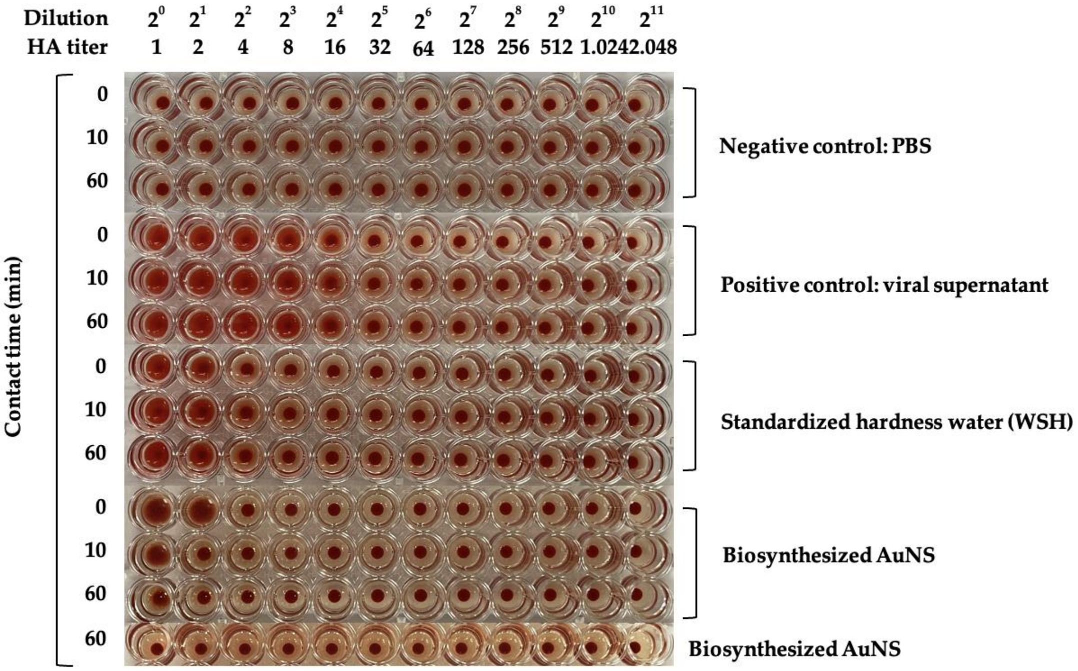


**Figure S5. Hemagglutination assay of influenza A H1N1 virus treated with nanostructures.** The HA titer of influenza A virus was evaluated in viral supernatant samples treated with standardized hardness water (WSH) and biosynthesized gold nanostructures (AuNS) using short (10 min) and long (60 min) contact times, followed by 1:2 dilution in 1X PBS. 1X PBS and nanostructure solution at 100 μg/mL (only for 60 min contact time) were used as negative controls. Untreated viral supernatant was used as a positive control.

**Table S1. Primers used in this study.** The primers designed to amplify genes encoding proteins with potential gold and copper-reducing activity, universal primers used as controls and for sequencing, and primers obtained from Spackman et al. for implementing the absolute quantification method via RT-qPCR are shown.

| Primer | Sequence 5′ → 3′ | Tm (°C) |
|---|---|---|
| trxB_F | ATAC**GCTAGC**ATGATGACTGAAGTAAATTA | 58,4 |
| trxB_R | ATC**AAGCTT**TGCTTCTTGCTTATCTTTTAATTC | 58,2 |
| cdr_F | **CACC**ATGCGTAAAATAATTGTAGTCGGT | 57,5 |
| cdr_R | TAAATCTCTAGCTTTGTAACCAATCAT | 52,7 |
| oxi_F | ATAC**GCTAGC**ATGATGAGTCATGATACAAATACTGTTG | 60,6 |
| oxi_R | ATC**AAGCTT**TCCGCTATACACCATCCCTAT | 60,7 |
| trmFO_F | ATAC**GCTAGC**ATGATGAGTCAAACTGTAAATATTG | 58,5 |
| trmFO_R | ATC**AAGCTT**AAGTGTTTTCTTATAATTCTCTAAATATTCGA | 57,2 |
| gor_F | ATAC**GCTAGC**ATGATGAGTCATGATACAAATACTGTTG | 57,7 |
| gor_R | ATC**AAGCTT**TCCGCTATACACCATCCCTAT | 57,6 |
| 16S_8F | AGAGTTTGATCCTGGCTCAG | 51,5 |
| 16S_1492R | ACGGCTACCTTGTTACGACTT | 50,1 |
| T7 terminador | GCTAGTTATTGCTCAGCGG | 53,4 |
| M+25_F | AGATGAGTCTTCTAACCGAGGTCG | 57,6 |
| M-124_R | TGCAAAAACATCTTCAAGTCTCTG | 53,9 |

**Table S2. Ct values and copy number obtained from qPCR amplification of the conserved segment of the M gene from the recombinant vector.** The table shows the Ct values obtained for each concentration of recombinant vector used (serial dilutions), along with the associated copy number values for each concentration. Ct values in bold are excluded from the method due to the lack of a linear relationship.

| Concentration (ng/μL) | Ct average | Copy number/μL | $Log_{10}$ copy number/μL |
|---|---|---|---|
| 0,152 | 7,30 | 1,38E+09 | 9,14 |
| 1,52E-02 | 10,52 | 1,38E+08 | 8,14 |
| 1,52E-03 | 13,21 | 1,38E+07 | 7,14 |
| 1,52E-04 | 16,40 | 1,38E+06 | 6,14 |
| 1,52E-05 | 19,19 | 1,38E+05 | 5,14 |
| 1,52E-06 | 22,53 | 1,38E+04 | 4,14 |
| 1,52E-07 | 26,72 | 1,38E+03 | 3,14 |
| 1,52E-08 | 29,68 | 1,38E+02 | 2,14 |
| 1,52E-09 | 32,52 | 1,38E+01 | 1,14 |
| **1,52E-10** | **33,59** | **1,38E+00** | **0,14** |
| **1,52E-11** | **34,45** | **1,38E-01** | **-0,86** |
| **1,52E-12** | **35,20** | **1,38E-02** | **-1,86** |

**Table S3.** Environmental bacterial isolates from Chilean, Peruvian, and Bolivian samples.

| Environmental Isolate | Origin | Optimal T (°C) |
|---|---|---|
| MF01 | Laguna del Maule | 37 |
| MF02 | Aislado clínico (Perú) | 37 |
| MF07 | Valle de la Muerte | 37 |
| MF10 | Salar de Uyuni (Bolivia) | 37 |
| MF15 | Laguna Cejar | 37 |
| MF16 | Laguna del Maule | 37 |
| MF17 | Laguna del Maule | 37 |
| MF18 | Mina El Teniente | 37 |
| MF19 | Mina El Teniente | 37 |
| MF20 | Mina El Teniente | 37 |
| BNF01 | Antártica | 37 |
| BNF05 | Antártica | 37 |
| BNF20 | Antártica | 25 |
| BNF22 | Antártica | 25 |
| BNF25 | Antártica | 25 |
| R37A | Antártica | 15 |
| M53 | Antártica | 15 |
| M53B | Antártica | 15 |

Each isolate was identified with a code of letters and numbers. The origin and optimal growth temperature for each isolate are shown.

**Table S4.** Genotype of the *E. coli* strains used.

| Strain | Main Characteristics | Source |
|---|---|---|
| *E. coli* BW25113 | *lacI*q, rrnBT14, Δ *lac*ZWJ16, *hsdR*514, Δ *araBADAH*33, Δ *rhaBADLD*78 | Datsenko y Wanner |
| *E. coli* TOP10 | $F^-$ *mcrA* Δ(*mrr-hsd*RMS-*mcr*BC)Φ80*lac*ZΔM15 Δ *lac*X74 *rec*A1 *ara*D139 Δ(*araleu*)7697 *gal*U *gal*K *rps*L ($Str^R$) *end*A1 *nup*G | Invitrogen® |
| *E. coli* BL21(DE3) | $F^-$ *omp*T *hsdS*$_B$ ($r_B^-$ $m_B^-$) gal *dcm rne*131 (DE3) | Invitrogen® |
